# Syncope and Epilepsy coexist in ‘possible’ and ‘drug-resistant’ epilepsy (Overlap between Epilepsy and Syncope Study - OESYS)

**DOI:** 10.1186/s12883-017-0822-5

**Published:** 2017-02-28

**Authors:** Andrea Ungar, Alice Ceccofiglio, Francesca Pescini, Chiara Mussi, Gianni Tava, Martina Rafanelli, Assunta Langellotto, Niccolò Marchionni, J. Gert van Dijk, Gianlugi Galizia, Domenico Bonaduce, Pasquale Abete

**Affiliations:** 1 Department of Clinical and Experimental Medicine, Syncope Unit, Geriatric Cardiology and Medicine, University of Florence, Florence, Italy; 2 Department of Neurological and Psychiatric Sciences, Epilepsy Center, University of Florence, Florence, Italy; 3 Geriatric and Gerontology Institute, University of Modena, Modena, Italy; 4 Geriatric Unit, Santa Chiara Hospital, Trento, Italy; 5 Division of Geriatrics, Ospedale “S. Maria di Ca’ Foncello”, Treviso, Italy; 6 Department of Neurology, Leiden University Medical Centre, Leiden, The Netherlands; 7 Istituti Clinici Scientifici Maugeri- Syncope unit – UOC Cure sub-acute, Milan, Italy; 8 Department of Translational Medical Sciences, University of Naples Federico II, Via S. Pansini, 80131 Naples, Italy

## Abstract

**Background:**

Differential diagnosis between syncope and epilepsy in patients with transient loss of consciousness of uncertain etiology is still unclear. Thus, the aim of the present work is to evaluate the prevalence of syncope in patients with “possible” or “drug-resistant” epilepsy.

**Methods:**

The Overlap between Epilepsy and SYncope Study (OESYS) is a multicenter prospective observational study designed to estimate the prevalence of syncope in patients followed in Epilepsy Centers for “possible” or “drug-resistant” epilepsy and assessed according the European Society of Cardiology (ESC) guidelines of syncope diagnosis.

**Results:**

One hundred seven patients were evaluated; 63 (58.9%) had possible and 44 (41.1%) drug-resistant epilepsy. A final diagnosis of isolated syncope was in 45 patients (42.1%), all with possible epilepsy (45/63, 71.4%). Isolated epilepsy was found in 21 patients (19.6%) and it was more frequent in the drug-resistant than in the possible epilepsy group (34.1% vs. 9.5%, *p* = 0.002). More importantly, syncope and epilepsy coexisted in 37.4% of all patients but the coexistence was more frequent among patients with drug-resistant than possible epilepsy (65.9% vs. 17.5%, *p* < 0.001).

**Conclusions:**

Isolated syncope was diagnosed in ≈ 70% of patients with possible epilepsy. Syncope and epilepsy coexisted in ≈ 20% of patients with possible and in ≈ 60% of patients with drug-resistant epilepsy. These findings highlight the need of ESC guidelines of syncope approach in patients with possible and drug-resistant epilepsy.

## Background

As many as 20–40% of patients diagnosed as having epilepsy may not actually have epilepsy [1]. In such patients, antiepileptic drugs (AEDs) are harmful because they have adverse effects, are ineffective, unnecessary, and reanalysis is postponed until their efficacy is judged, usually after a very long time [1, 2]. Syncope is the most frequent cause of misdiagnosis in epilepsy [2]. In patients defined as having “drug-resistant epilepsy”, attacks persist because the underlying disorder (i.e., syncope) has not been correctly diagnosed [3]. There are several reasons why syncope may be erroneous considered as epileptic seizures. Firstly, syncope affects up to 40% of the population [4], so even a small proportion of cases may contaminate “epilepsy” cohorts. Secondly, syncope is frequently associated with abnormal movements such as myoclonic jerks, oral automatism, head-turning and, more rarely, urinary incontinence, thus mimicking the clinical presentation of epileptic seizures [2, 5–7]. Thirdly, syncope and seizures may coexist in a patient, either by pure chance or by pathophysiology mechanism. Temporal seizure may, on rare occasions cause asystole, and therefore, syncope by cardiac mechanism [8–10]. Conversely, syncope may provoke a true epileptic seizure [11, 12]. More specifically, an epileptic-anoxic seizure arising usually from a temporal lobe is seen in epilepsy; whereas an anoxic-epileptic seizure (an epileptic seizure triggered by syncope, typically during recovery) is seen in syncope in patients without necessarily having epilepsy.

Thus, the rate of coexistence of epileptic seizures and syncope appears complex and needs to be better defined.

The Overlap of Epilepsy and SYncope Study (OESYS) is multicenter prospective observational study designed to estimate the prevalence of syncope according to the European Society of Cardiology (ESC) guidelines [4] in patients followed in Epilepsy Centers for possible or drug-resistant epilepsy.

## Methods

This study was carried out on consecutive patients followed in Epilepsy Centers for possible or drug-resistant epilepsy and evaluated in 4 different Italian Syncope Units (Florence, Modena, Trento and Naples) between November 2009 and June 2012.

### Inclusion and exclusion criteria

Patients were selected in the Epilepsy Centers by neurologists if they had a diagnosis of either possible or drug-resistant epilepsy and presented recurrent episodes of non-convulsive transient loss of consciousness (T-LOC) followed or not followed by jerks or involuntary movements, whose origin remained unknown after the neurological clinical and diagnostic evaluations. All episodes of functional T-LOC (non-epileptic T-LOC with normal blood pressure and heart rate) were classified as Psychogenic Non-Epileptic Seizures (PNES).

Thus, the inclusion criteria were age ≥ 18 years, recurrent T-LOC (≥2 episodes) of unknown cause and:

- drug-resistant epilepsy, defined according to the International League Against Epilepsy (ILAE) Commission as “failure of adequate trials of two tolerated and appropriately chosen and used AED schedules (whether as mono-therapy or in combination) to achieve sustained seizure freedom”. Seizure-free duration that is at least three times the longest interseizure interval prior to starting a new intervention would need to be observed or at least 12 months [13].

or

b) possible epilepsy, defined as “seizure with an alternative explanation for the attack and insufficient evidence to support a confident diagnosis of epilepsy” [14].

The exclusion criteria were the presence of generalized tonic-clonic seizure (GTCS) and the inability or unwillingness to give informed consent.

### Management strategy

In the Epilepsy Centers the evaluation of the patients included: i) history, mainly focused on past and current AEDs treatment, comorbidity with neurological and non neurological diseases, clinical aspects of described and/or witnessed episodes (i.e. number, type, predisposing circumstances, prodromes, etc); ii) physical examination; iii) instrumental tests, including electroencephalogram (EEG) and computed tomography (CT) or magnetic resonance imaging (MRI) of the brain.

Selected patients, who fulfilled the inclusion criteria were referred to the Syncope Unit and managed according to the ESC guidelines for diagnosis and management of syncope^12^. The initial evaluation consisted of a careful history, focused on cardiovascular diseases and drugs, and patient tests that included a 12-lead electrocardiogram, orthostatic blood pressure measurements, Head-Up Tilt testing (HUT) with sublingual nitroglycerin according to the Italian protocol [15], and Carotid Sinus Massage (CSM), performed according to the symptoms method [16]: when associated with reproduction of spontaneous symptoms by patients or relatives, HUT and CSM were defined as diagnostic of syncope. Possible cardiac causes of syncope were evaluated using previous medical history, drug use and standardized cardiovascular evaluation when indicated. In patients with unexplained syncope, a loop recorder (ILR) was implanted for diagnosis at the end of an otherwise negative work-up.

The final diagnosis was made though consensus between a syncope expert and an epileptologist at the end of evaluation and confirmed during follow-up, conforming to the ESC classification^12^. In particular, a) patients “positive” to syncope algorithm were considered as “isolated syncope”, b) patients “negative” to syncope algorithm were considered as “isolated epilepsy” after a careful consensus between a syncope expert and an epileptologist, and c) in patients “positive” to syncope algorithm and with suggestive clinical evidences of epilepsy, the coexistence of syncope and epilepsy was considered. New findings were treated appropriately.

### Follow-up

Follow-up visits were planned at 3, 6 and 12 months during which data was collected based on a predefined structured questionnaire…

### Statistical analysis

The sample size of the study (*n* = 100) was calculated by assuming prevalence of coexistence of syncope and epilepsy equal to 40% in a population of possible and drug-resistant epilepsy (95% confidence interval of 30–50%). Statistical analysis was performed using Statistica version 8.0 (Stat Soft Italia, Padova, Italy). Student’s *t*-test for unpaired data was used to compare differences in continuous data between groups. The chi-square test was used for dichotomous variables. Anova and Bonferroni’s post-hoc test were performed to compare mean in more than two groups. A value of *p* < 0.05 was considered significant. Data was reported as mean ± standard deviation or as percentages.

## Results

Out of 4800 consecutive patients followed in the Epilepsy Centers 107 (2.2%) (46 men, 61 women, mean age 56 ± 21 years) presented recurrent T-LOC of unknown cause and a diagnosis of possible or drug-resistant epilepsy 63 patients (58.9%) had possible epilepsy and 44 (41.1%) drug-resistant epilepsy (Table 1). Patients with drug-resistant epilepsy had a significantly higher frequency of heart disease and intake of cardiovascular drugs than those with possible epilepsy (50 vs. 20.6% and 56.8 vs 33.3, respectively). Seventy-seven patients (72.0%) used AEDs distributed as follows: all patients with drug-resistant epilepsy and more than half patients with possible epilepsy (Table 1). The median number of T-LOC episodes for patient in the last year was 4 ± 4 (range 2–20) and 66 patients had pre-syncopal symptoms (61.7%). After T-LOC, involuntary movements, including myoclonic jerks, were present in half of the patients (54.2%). The most frequent after T-LOC event was mental confusion (24.3%) and half the number of patients (51.4%) had suffered physical injury during the episode (Table 1).

**Table 1:** Baseline characteristics of patients

	Total population (*n* = 107)	Possible Epilepsy (*n* = 63, 58.9%)	Drug- resistant Epilepsy (*n* = 44, 41.1%)
Mean age, years (mean ± SD, range)	56 ± 21 (18–88)	52 ± 21 (18–88)	62 ± 18 (29–88)
Male gender, n (%)	46 (43.0)	28 (44.4)	18 (40.9)
Heart diseases^a^, n (%)	35 (32.7)	13 (20.6)	22 (50.0)
Neurological diseases^b^, n (%)	32 (29.9)	22 (34.9)	10 (22.7)
Cardiovascular drugs^c^, n (%)	46 (43.0)	21 (33.3)	25 (56.8)
Antiepileptic drugs^d^, n (%)	77 (72.0)	33 (52.4)	44 (100.0)
T-LOC/patient/year (mean ± SD, range)	4 ± 4 (2–20)	3 ± 4 (2–20)	4 ± 4 (2–20)
T-LOC Prodromal symptoms	66 (61.7)	40 (63.5)	26 (59.1)
After T-LOC characteristics			
Involuntary movements	58 (54.2)	37 (58.7)	21 (47.7)
Mental contusion, n (%)	26 (24.3)	26 (41.3)	12 (27.3)
Physical injury, n (%)	55 (51.4)	31 (49.2)	24 (54.6)
Comorbidities, n (%)			
Hypertension, n (%)	41 (38.3)	20 (31.7)	21 (47.7)
Diabetes, n (%)	10 (9.3)	6 (9.5)	4 (9.1)
Dyslipidemia, n (%)	18 (16.8)	9 (14.3)	9 (20.5)

*T-LOC* transient loss of consciousness

^a^Ischemic cardiomyopathy, atrial fibrillation, pulmonary embolism, heart failure

^b^Stroke, Parkinson’s disease, dementia, limbic encephalitis, normal pressure hydrocephalus

^c^Diuretics, angiotensin-converting enzyme inhibitors, angiotensin II receptor blockers, calcium channel blockers, nitrate, alpha-blockers, beta-blockers, antiarrhythmics, cardiac glycosides

^d^Phenobarbital, phenytoin, lamotrigine, valproate, levetiracetam, carbamazepine, gabapentin, pregabalin, topiramate, primidone, vigabatrin

EEG and neuro-imaging results are shown in Table 2. Normal or non-epileptiform abnormalities were common and more frequent in the possible epilepsy group than in the drug-resistant group. Interictal epileptiform activity was present in more than 70% of the patients with drug- resistant epilepsy. Brain CT/MRI did not show abnormalities in 58 patients (54.2%), leukoencephalopathy was present in 24 (22.4%) and cortical atrophy in 9 (8.4%) patients.

**Table 2:** EEG and neuroradiological (CT/MRI) findings

	Total population (*n* = 107)	Possible Epilepsy (*n* = 63, 58.9%)	Drug-resistant Epilepsy (*n* = 44, 41.1%)
EEG: normal pattern^a^, n (%)	12 (11.2)	12 (19.0)	0 (0)
EEG: abnormal not epileptiform^b^, n (%)	61 (57.0)	51 (81.0)	10 (22.7)
EEG: epileptiform^c^, n (%)	34 (31.8)	0 (0.0)	34 (77.3)
Temporal lobe spike activity, n (%)	27 (25.2)	0 (0.0)	27 (61.4)
Neuro-imaging (CT/MRI)			
Normal, n (%)	58 (54.2)	40 (63.5)	18 (40.9)
Tumors, n (%)	4 (3.7)	3 (4.8)	1 (2.3)
Cortical Atrophy, n (%)	9 (8.4)	5 (7.9)	4 (9.1)
Leukoaraiosis, n (%)	24 (22.4)	14 (22.2)	10 (22.7)
Neurosurgery findings, n (%)	7 (6.5)	0 (0.0)	7 (15.9)
Cortico-subcortical infarcts, n (%)	2 (1.9)	0 (0.0)	2 (4.5)
Limbic Encephalitis findings, n (%)	1 (0.9)	0 (0.0)	1 (2.3)
Cortical malformations, n (%)	2 (1.9)	1 (1.6)	1 (2.3)

*CT* computed tomography, *EEG* electroencephalogram, *MRI* magnetic resonance imaging

^a^Background activity generally characterized by alpha rhythm (with a frequency of 8–13 Hz), reacting to the opening and closing of the eyes, and a typical posterior representation; morphology mostly regular

^b^Slow activity (theta activity) focal or diffuse, non-paroxysmal and/or non-dominant

^c^Epileptiform activity (spikes; polyspikes, sharp waves, spikes and waves or polyspike-waves complexes) both generalized and focal

Orthostatic hypotension was present in 33 patients (30.8%) (Table 3). HUT reproduced vasovagal syncope in 52 patients (48.6%), of which 18 patients had myoclonic jerks that resulted frequent in patients with possible than in drug-resistant epilepsy (25.4% vs. 4.5%). Carotid Sinus message was diagnostic for carotid sinus syndrome in 7 patients (6.5%). Based on suspicion of a cardiac syncope, 43 patients (40.2%) underwent second-level cardiac examinations (echocardiography, 24 h Holter monitoring, and exercise test) that revealed pathological conditions in 3 cases (n.1 bradycardia/tachycardia syndrome, n. 1 advanced second-degree atrio-ventricular block, n.1 severe aortic stenosis). Thirteen patients (12.1%) received an ILR that lead 3 diagnoses during follow-up (n.1 ventricular tachycardia and n.2 asystolic pauses) (Table 3).

**Table 3:** Cardiovascular and neurally-mediated diagnostic tests

	Total population (*n* = 107)	Possible Epilepsy (*n* = 63)	Drug-resistant Epilepsy (*n* = 44)
Abnormal ECG^a^, n (%)	9 (8.4)	6 (9.5)	3 (6.8)
Orthostatic Hypotension, n (%)	33 (30.8)	17 (27.0)	16 (36.4)
Head-up Tilt testing			
Performed, n (%)	92 (86.0)	55 (87.3)	37 (84.1)
Diagnostic, n (%)	52 (48.6)	35 (55.6)	17 (38.6)
Myoclonic jerks, n (%)	18 (16.8)	16 (25.4)	2 (4.5)
Carotid Sinus Massage			
Performed, n (%)	104 (97.2)	60 (95.2)	44 (100.0)
Diagnostic, n (%)	7 (6.5)	5 (7.9)	2 (4.5)
Echocardiography performed ^b^, n (%)	43 (40.2)	27 (42.9)	16 (36.4)
24 h Holter monitoring performed^c^, n (%)	43 (40.2)	20 (31.7)	23 (52.3)
Exercise test performed, n (%)	13 (12.1)	10 (15.9)	3 (6.8)
Electrophysiological study performed, n (%)	3 (2.8)	2 (3.2)	1 (2.3)
ILR implanted ^d^, n (%)	13 (12.1)	6 (9.5)	7 (15.9)

*ECG* electrocardiogram, *ILR* intermittent loop recorder

^a^Left bundle-branch block, bifascicular block, previous myocardial infarction, atrial fibrillation

^b^Revealed 1 severe aortic stenosis

^c^Revealed 1 bradycardia/tachycardia syndrome and 1 advanced second-degree AV block

^d^Revealed 1 ventricular tachycardia and 2 asystolic pauses

**Fig. 1:**
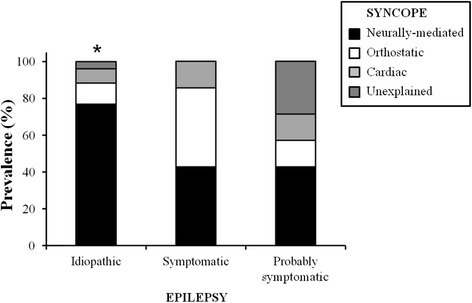
Prevalence of different type of syncope and epilepsy in patients with coexistence of syncope and epilepsy. (*p < 0.01 neurally-mediated vs. patients with symptomatic and probably symptomatic epilepsy)

The diagnoses at the end of the work-up in the syncope unit are shown in Table 4. Isolated syncope was diagnosed in 45 patients (42.1%), all of them being patients enrolled for possible epilepsy (71.4% of the group). The most frequent cause of isolated syncope was neurally-mediated (28.0%), while cardiac syncope was rare (1.9%). In 2 patients (2.8%) the episodes were strongly suggestive of syncope, but the etiology remained unexplained (Table 4). Isolated epilepsy was diagnosed in 21 patients (19.6%), of which 15 presented with drug-resistant epilepsy (34.1%) and only 6 with possible epilepsy (Fig. 1). Isolated epilepsy was classified as idiopathic in 14 patients (13.1%), symptomatic in 6 patients (5.6%) and probably symptomatic only in 1 patient (0.9%) (Table 4).

**Table 4:** Diagnosis at the end of work-up in the Syncope Unit

	Total population (*n* = 107)	Possible Epilepsy (*n* = 63)	Drug-resistant Epilepsy (*n* = 44)
Isolated Syncope, n (%)	45 (42.1)	45 (71.4)	0 (0.0)
Neurally-mediated, n (%)	30 (28.0)	30 (47.6)	0 (0.0)
Vasovagal, n (%)	24 (22.4)	24 (38.1)	0 (0.0)
Carotid Sinus Syndrome, n (%)	5 (4.7)	5 (7.9)	0 (0.0)
Situational, n (%)	1 (0.9)	1 (1.6)	0 (0.0)
Orthostatic hypotension, n (%)	10 (9.3)	10 (15.9)	0 (0.0)
Cardiac, n (%)	2 (1.9)	2 (3.2)	0 (0.0)
Unexplained syncope, n (%)	3 (2.8)	3 (4.8)	0 (0.0)
Isolated Epilepsy, n (%)	21 (19.6)	6 (9.5)	15 (34.1)
Idiopathic, n (%)	14 (13.1)	5 (7.9)	9 (20.5)^a^
Symptomatic, n (%)	6 (5.6)	1 (1.6)	5 (11.4)
Probably symptomatic, n (%)	1 (0.9)	0 (0.0)	1 (2.3)
Syncope & Epilepsy	40 (37.4)	11 (17.5)	29 (65.9)
Psychogenic non-epileptic seizures	1 (0.9)	1 (1.6)	0 (0.0)

^a^In 2 of 9 patients with idiopathic isolated epilepsy, psychogenic non-epileptic seizures are also present

Syncope and epilepsy coexisted in 37.4% of all patients but the coexistence was more prevalent in drug-resistant than in possible epilepsy (65.9% vs. 17.5%) (Table 4). In Fig. 1, the frequency of the different types of syncope and epilepsy is shown in patients with coexistence of syncope and epilepsy. Patients showing idiopathic epilepsy presented the highest percentage of neurally-mediated syncope (76.9%).

PNES have been diagnosed in only 1 patient with possible diagnosis of epilepsy and coexisted in 2 of 9 patients with idiopathic isolated epilepsy.

### Follow-up

Forty-seven patients with an initial diagnosis of possible epilepsy and 38 with drug-resistant epilepsy were available for follow-up analysis (85 of the enrolled patients, 79.4%) (Mean follow-up duration was 390 days, range 3 months–3.5 years). Forty patients (47.0%) had a recurrence of T-LOC (mean number of episodes was 4 ± 3, range 1–20); of these, 13 had recurrence of syncope (32.5%), 22 of epileptic seizures (55.0%) and 5 of both (12.5%). Regarding AEDs, 32 patients with possible epilepsy were on AEDs before enrolment (32/47, 68%), in 21 patients syncope was diagnosed and AEDs were discontinued (21/38, 55.2%). In 11 patients with possible epilepsy at enrollment and in whom the diagnosis of epilepsy was confirmed, AEDs was continued in 11 (11/47, 23.4%), and started in 8 (8/47, 17.0%).

## Discussion

A group of highly selected patients (2.2% of the full amount of patients followed in the Epilepsy Centers) who presented recurrent T-LOC of unknown cause when evaluated by the neurologists. Patients with a diagnosis of possible or drug-resistant epilepsy were enrolled and referred to the syncope Units. Isolated syncope was diagnosed in the 42.1% of all cases, being more frequent among patients affected by possible epilepsy (≈70%). Interestingly, syncope and epilepsy coexisted in ≈ 40% of patients. In the follow-up, T-LOC recurrence was ≈ 50%. More importantly, in patients with possible epilepsy taking AEDs before enrolment (≈50%) the administration was discontinued, confirmed, and started according to epilepsy diagnosis confirmation. These results involve important implications for the management of T-LOC patients with possible or drug-resistant epilepsy.

### Overlap of syncope and epilepsy

Few studies addressed the overlap between syncope and epilepsy [1, 17, 18]. Although the selection of patients was very restrictive (2.2% of consecutive patients followed in the Epilepsy Centers) OESYS shows a very high occurrence of syncope, i.e. ≈70% of patients with possible epilepsy in the absence of witnessed and/or epileptiform EEG abnormalities. Previous studies included patients with either recurrent “seizure-like” episodes [1] or presented “episodes of loss of consciousness, falls and seizures” [16]. In contrast, in OESYS, the main selection symptom was the “T-LOC”. In some forms of epilepsy, postural control remains intact, and therefore, they do not determine T-LOC and are not confused with syncope [19]. Consequently, in selected patients a probability of having syncope is very high. Moreover, the inclusion of patients with “suspected epilepsy” might have also caused the enrolment of patients without epilepsy. In OESYS, only 52% of patients with possible epilepsy were on AEDs treatment. These patients were selected by neurologists, highly experienced in the management of epilepsy and trained in the diagnosis of syncope, and therefore, with clinical features poorly suggestive of epilepsy. Another aspect to consider is that patients in our study had a higher mean age in comparison with previous series (56 vs. 39 and 41 years) [1, 17]. Our patients presented a high comorbidity for heart diseases and most of them were on therapy with cardiovascular drugs (no data available in the previous studies). Considering the high prevalence of syncope in subjects over 65 years (from 35 to 39%) [1, 17], and the higher recurrence of syncope among patients with cardiovascular comorbidity [20, 21], the high frequency of syncope in our sample could, in part, be due to these demographic and clinical characteristics.

### Coexistence of syncope and epilepsy

In our study, the coexistence of syncope and epilepsy was found in ≈ 40% of patients and in more than 60% of patient with drug-resistant epilepsy. Differently from the “possible epilepsy” group, no patient with drug resistant epilepsy was found with isolated syncope. This could implicitly confirm the presence of epilepsy in this group, in some cases, coexisting with syncope. Rangel et al. showed the coexistence of syncope and epilepsy only in 21% of the patients with refractory epilepsy [18]. As suggested before, the higher prevalence observed in our study may be due to a higher mean age with respect to those patients studied by Rangel et al. [18]. In addition, neurally-mediated syncope together with idiopathic epilepsy represents the more frequent association (50%). However, idiopathic epilepsy is a young person condition. Neurally mediated syncope has onset in adolescence, with a second incidence peak in the advancing age [22]. Similarly, idiopathic epilepsy is a young person condition but it may have also “a late onset” because of a genetic predisposition triggered by acquired epileptogenic factors [23]. Interestingly, it has been suggested that autonomic seizures may depend on age-dependent epileptogenic susceptibility (Panayiotopoulos syndrome) [24]. Nevertheless, neurally-mediated syncope is the more frequent syncope observed in a geriatric sample (66%) [25]. Thus, it should be hypothesized that the higher frequency of coexistence of syncope and epilepsy observed in our study may have the same autonomic dysfunction origin.

### Epilepsy misdiagnosis

It should be underlined that at the end of the diagnostic work-up, the presumptive diagnosis of epilepsy was confirmed only in 10% of patients for possible epilepsy without epileptiform EEG abnormalities. For our sample the inter-ictal EEG was used even if its sensitivity is limited, ranging between 26 and 59% [26]. This tool was chosen in order to allow a high number of epileptic patients referred to the Epilepsy Centers including false positive patients in whom a final diagnosis of epilepsy was not confirmed. Moreover, these patients had T-LOC episodes often not recalled and/or occurred in the absence of witnesses leading to an increase of the possibility of the clinical diagnosis. Rodrigues et al. excluded patients with brain lesions [17] while in OESYS secondary forms of epilepsy were not excluded. Thus, abnormalities on neuro-imaging, occurring in about half of patients with possible epilepsy, might have influenced the neurologists to consider epilepsy more than the non-specific clinical presentation and normal EEG features would otherwise suggest.

Many authors have assessed the importance of the clinical presentation of T-LOC in distinguishing syncope from epilepsy [27, 28]. In our study, involuntary movements during the T-LOC were referred in almost half of patients with possible epilepsy and, more importantly, myoclonic jerks occurred in a high proportion of patients with possible epilepsy during the HUT-evocated T-LOCs. Accordingly, “convulsive syncope” is often characterized by involuntary movements, mostly myoclonic jerks [29]. The high frequency of myoclonic jerks may have also contributed to a selection bias, and epileptic phenomena might be misdiagnosed at the initial evaluation. Interestingly, in this group, T-LOC episodes manifested with the same stereotyped features while T-LOC differed from the usual seizures in the group of patients with drug-resistant epilepsy. The data supports the high frequency of coexisting epilepsy and syncope in the group with drug-resistant epilepsy (65.9%), and highlight the importance of a careful clinical characterization of T-LOC episode that requires a careful knowledge of signs and symptoms of syncope and epilepsy.

Finally, PNES represent a serious diagnostic challenge for physicians, especially in drug-resistant epilepsy. Video–electroencephalography studies have provided detailed knowledge of the spectrum of visible PNES manifestations. Unfortunately, in our study video-electroencephalography was not performed. Moreover, findings based on the self-report of patients with well-characterized PNES and witnesses of their seizures demonstrate a large intra- and inter-individual variability of reported PNES manifestations that may lead to incorrect diagnoses [6]. PNES accounts for 20 to 30% of patients seen in epileptic clinics [30–32]. Differently, in our sample PNES has been found only in 1 patient with possible diagnosis of epilepsy and coexisted in 2 of of 9 patients with idiopathic isolated epilepsy. However, we should consider that our sample is made up by patients with T-LOC of unknown cause, selected among 4800 subjects followed in the Epilepsy Center. Thus, patients with PNES could have been likely diagnosed and not carried out for our study because their cause of T-LOC was already clear. Moreover, our HUT procedure did not include scalp EEG, as this is the best way to identify functional T-LOC.

### Diagnostic and therapeutic implications

In more than 70% of patients with possible epilepsy, the final diagnosis of epilepsy was not confirmed. It should be underlined that pharmacological treatment with AEDs was undertaken only in half of these patients because of the possibility of the initial diagnosis. At the end of the evaluation, AEDs were discontinued in more than 30% of patients suggesting a high percentage of true misdiagnosis of epilepsy in our study. This data also confirms that in clinical practice AEDs should be started when the diagnosis is definite [2]. Our diagnostic protocol provided the ILR implantation for selected patients with a high suspicion of cardiogenic and/or unknown syncope. It has been reported the use of ILR in a small cohort of syncopal patients able to identify an arrhythmogenic cause at the origin of seizure-like manifestations [33].

Finally, is “OESYS” approach helpful to define the presence of syncope in patients with possible or drug-resistant epilepsy? Angus-Leppan described that in 158 patients with loss of consciousness or possible epilepsy, the neurologist reached a diagnosis in 87% of the cases (43% epilepsy, 25% syncope, 12% non-epileptic seizures and in 7% other diagnoses). Unfortunately, in 13% of the cases the diagnosis remained unknown [5]. In this subset of patients, the “OESYS” approach may be particularly helpful.

### Limitation of the study

The main limitation of OESYS study is the absence of the data regarding patients with “definite” epilepsy. This lack is clearly related to the inclusion criteria of the study. Only patients with “possible or “drug-resistant” epilepsy followed in the Epilepsy Centers were enrolled. Of course, the retrospective recovery of this data is unreliable especially for the mix of clinical centers, and the vagueness of the groups’ composition. Thus, although patients with clear diagnosis of epilepsy (not enrolled in our study) may be easier recognized, the detection of syncope in patients with “possible” or “drug resistant” epilepsy diagnosis may be extremely difficult. In his regard, OESYS’ protocol should be extremely helpful especially in patients with uncertain epilepsy in whom the clinical scenario is unclear. A further limitation is the lack of video-EEG monitoring especially in the diagnosis of PNES. However, as our sample has been selected from a population deeply studied in the Epilepsy Centers, we could expect that from this group, already diagnosed as PNES, only a very low percentage was enrolled in our study.

## Conclusions

Syncope was diagnosed in ≈ 70% of patients initially identified with “possible” epilepsy. It means that through diagnostic algorithm a clear diagnosis of syncope was found out despite of the initial suspect of “possible epilepsy”. Syncope and epilepsy coexisted in ≈ 40% of patients with “possible” and “drug-resistant” epilepsy. Syncope recurrence was ≈ 50% in the follow-up. AEDs administration in patients with “possible” epilepsy was started, stopped or continued, according to syncope diagnosis. Thus, diagnostic protocol for syncope plays a key role in the management of T-LOC patients with “possible” or “drug-resistant” epilepsy.

## Abbreviations

AEDs: Antiepileptic drugs
CSM: Carotid Sinus Massage
CT: Computed tomography
EEG: Electroencephalogram
ESC: European Society of Cardiology
HUT: Head-Up Tilt testing
ILR: Implantable loop recorder
MRI: Magnetic resonance imaging
OESYS: Epilepsy and SYncope Study
PNES: Psychogenic Non-Epileptic Seizures
T-LOC: Transient loss of consciousness
